# Image-Based Computational Cardiology: From Data to Understanding

**DOI:** 10.1155/2014/120960

**Published:** 2014-06-01

**Authors:** Linwei Wang, Vicky Y. Wang, Heye Zhang

**Affiliations:** ^1^Rochester Institute of Technology, Rochester, NY 14634, USA; ^2^Auckland Bioengineering Institute, The University of Auckland, Auckland 1010, New Zealand; ^3^Shenzhen Institutes of Advanced Technology, Shenzhen 518055, China

Over the past decade, rapid advances in imaging technologies have enabled state-of-the-art image-based analysis of cardiac function at a variety of scales and from multiple aspects, such as cardiac electrophysiology, cardiac biomechanics, and cardiovascular system. This special issue aims to showcase developments in cardiac image computing over the past five years, with a special emphasis given to novel computing techniques used to analyze cardiac anatomy and functions for better understanding of cardiac diseases. The final issue includes 8 high quality manuscripts that addressed some very interesting and timely research questions faced by researchers worldwide. The topics range from the improvement of real-time imaging acquisition, through the design of novel image analysis techniques and image-guided surgical planning tools, to cardiovascular risk assessment and detection.

Studying the evolution of heart formation has become an active research area [1]. Embryonic heart morphogenesis (EHM) is a complex and dynamic process where the heart transforms from a single tube into a four-chambered pump. Conventional imaging techniques fail to capture this process due to limited penetration depth. H. Mao et al. utilized confocal microscopy imaging with tissue optical immersion clearing technique to image the heart at different stages of development for an EHM study of quail. This area of study not only showed promising results in obtaining snapshots of hearts during growth, but also laid the foundation to investigate data-driven modeling of heart growth, which in turn could deepen our understanding of the cause, progression, treatment, and prevention of diseases related to cardiac growth.

Improving the speed of image reconstruction directly enhances the abundance of the data. In the work of N. Cai et al., a new method was developed to improve compressive sensing (CS) based reconstruction for dynamic cardiac MRI leveraging the theory of structured sparse representation. Their experimental results demonstrated that the proposed method improved the reconstruction quality of dynamic cardiac cine MRI over the state-of-the-art CS method [2].

Whilst numerous researches have been dedicated to imaging cardiac structure and mechanical function, noninvasive imaging of cardiac electrical activities remains to be a challenging task which entails the use of sophisticated mathematical models and optimization techniques [3, 4]. A. Rahimi et al. presented the superiority of an Lp-norm model over its L1 and L2 counterparts in imaging cardiac current sources with various spatial extents and distributions. Through computer-simulated and real-data experiments, they further demonstrated the feasibility of the proposed method in imaging the complex structure of excitation wavefront, as well as current sources distributed along the postinfarction scar border—the region that plays a critical role in the triggering and sustaining of lethal arrhythmias.

Extracting anatomical and physiological information of the heart from cardiac images requires robust yet simple segmentation techniques that suit different application purposes and different image qualities [5]. The work of J. Bayer et al. attempted to fit continuous parametric surfaces to scattered geometric data points forming frontiers delimiting physiologic structures in segmented images, and they successfully verified their methods by reconstructing a geometric model of a mouse's ventricles from a CT scan. This type of segmentation technique provides useful means of visualizing anatomical structures from low-quality images. C. Zhong et al. also investigated model-based image segmentation technique to reconstruct coronary artery (CA) segments that correspond to myocardial segments typically shown in conventional echocardiographic sectional images. By incorporating the existing Chinese visible human (CVH) data and 5 sets of clinical CT data, they established 3D CA models and studied their application in localization of CA segments. The fully reconstructed 3D model allowed virtual dissection to simulate convectional sections of transthoracic echocardiography. A preliminary study involving 170 patients was also undertaken to assess their proposed technique.

With coronary artery disease (CAD) becoming one of the most frequent causes of heart failure, there continues to be an urgent need to design prompt and accurate clinical diagnosis for CAD [6]. For example, noncalcified plaques (NCPs) on coronary artery trees are associated with the presence of lipid-core plaques that are prone to rupture. Thus, it is important to detect and monitor the development of NCPs. Y. Li et al. proposed a mathematical morphological approach to quantitatively analyze the noncalcified plaques on a three-dimensional coronary artery wall model (3D-CAWM). This work combined Voxel-Map analysis techniques, plaque locating, and anatomical location related labeling, which was demonstrated to achieve more detailed and comprehensive coronary tree wall visualization.

Image-guided surgery has now become one of the most active research topics in cardiac imaging computing [7]. Y. Wu et al. reconstructed a three-dimensional digitized visible model of human thoracic structures to provide morphological data for imaging diagnosis and thoracic-cardiovascular surgery. This research provided an efficient learning platform for medical students or junior surgeons to better interpret human thoracic anatomy and practice virtual thoracic and cardiovascular surgery.

This special issue concludes with a clinical study that examined the relationship between blood pressure variation (BPV) and carotid intima-media thickness (IMT) for 60 subjects (aged between 33 and 79) in Shenzhen. H. Xiong et al. found that both the daytime systolic BPV and 24-hour systolic BPV evaluated by three indices are positively associated with IMT. They conclude that daytime systolic BPV is the best variable to represent the increasing of carotid IMT. In addition, after adjusting by age, sex, smoking, hypertension, and mean blood pressure (BP) and peak-to-peak values, 24 h diastolic BPV evaluated with standard deviation also presents the favorable performance.

Overall, the 8 manuscripts included in this special issue provide a sample of the current state in image-based computational cardiology, including the classic topics such as image segmentation and image reconstruction, the relatively young areas such as growth modeling and electrophysiological imaging, and the use of computational techniques in image-based understanding, diagnosis, and surgery planning of cardiac diseases. The continuing effort in all these expanding and interconnected areas will likely improve our understanding of the mechanism, treatment, and prevention of cardiac diseases.
